# Ethephon induced abscission in mango: physiological fruitlet responses

**DOI:** 10.3389/fpls.2015.00706

**Published:** 2015-09-15

**Authors:** Michael H. Hagemann, Patrick Winterhagen, Martin Hegele, Jens N. Wünsche

**Affiliations:** Section Crop Physiology of Specialty Crops, Institute of Crop Science, University of HohenheimStuttgart, Germany

**Keywords:** ethylene receptors, fruitlet abscission zone, fruitlet pedicel, fruitlet pericarp, gene expression, polar auxin transport capacity, soluble carbohydrates

## Abstract

Fruitlet abscission of mango is typically very severe, causing considerable production losses worldwide. Consequently, a detailed physiological and molecular characterization of fruitlet abscission in mango is required to describe the onset and time-dependent course of this process. To identify the underlying key mechanisms of abscission, ethephon, an ethylene releasing substance, was applied at two concentrations (600 and 7200 ppm) during the midseason drop stage of mango. The abscission process is triggered by ethylene diffusing to the abscission zone where it binds to specific receptors and thereby activating several key physiological responses at the cellular level. The treatments reduced significantly the capacity of polar auxin transport through the pedicel at 1 day after treatment and thereafter when compared to untreated pedicels. The transcript levels of the ethylene receptor genes *MiETR1* and *MiERS1* were significantly upregulated in the pedicel and pericarp at 1, 2, and 3 days after the ethephon application with 7200 ppm, except for *MiETR1* in the pedicel, when compared to untreated fruitlet. In contrast, ethephon applications with 600 ppm did not affect expression levels of *MiETR1* in the pedicel and of *MiERS1* in the pericarp; however, *MiETR1* in the pericarp at day 2 and *MiERS1* in the pedicel at days 2 and 3 were significantly upregulated over the controls. Moreover, two novel short versions of the *MiERS1* were identified and detected more often in the pedicel of treated than untreated fruitlets at all sampling times. Sucrose concentration in the fruitlet pericarp was significantly reduced to the control at 2 days after both ethephon treatments. In conclusion, it is postulated that the ethephon-induced abscission process commences with a reduction of the polar auxin transport capacity in the pedicel, followed by an upregulation of ethylene receptors and finally a decrease of the sucrose concentration in the fruitlets.

## Introduction

Plant organ shedding or abscission is a highly coordinated process governed by the interplay of several plant metabolites, in particular phytohormones, carbohydrates, and polyamines (Sexton and Roberts, 1982; Malik and Singh, 2003; Xie et al., 2013). Abscission can be initiated in response to disease pressure, pest injury, or climate extremes, leading to interorgan competition for assimilates (Patterson and Bleecker, 2004; Botton et al., 2011). Understanding the regulation of genes encoding for proteins involved in synthesis, perception, and transport of these abscission relevant metabolites is of paramount importance for increasing the productivity of horticultural crops. This fundamental knowledge can be specifically utilized for devising practical solutions, ranging from marker-assisted genotype selection to crop management strategies using for example effective and growth stage dependent irrigation strategies and applications of plant growth regulators (Estornell et al., 2013).

Fruit drop is a yield-limiting factor for the production of several specialty crops, for example sweet cherry (Blanusa et al., 2005), litchi (Kuang et al., 2012), or mango (Singh et al., 2005). Of particular concern in many mango production systems worldwide is the extensive fruitlet drop. This major production constraint has been extensively studied at the orchard level (Singh et al., 2005) and was also a key research objective by Hagemann et al. (2014) who investigated the potential use of plant growth regulators, irrigation techniques, and cropping systems for improving fruit retention in mango. Both, biotic and abiotic factors have been frequently suggested as the key triggers for inducing fruitlet drop in mango (Singh et al., 2005). Biotic factors are mainly the lack of pollination or fertilization of flowers and pest or disease pressure that subsequently lead to seed degeneration (Singh and Arora, 1965). Abiotic factors associated with fruitlet drop are extensive drought periods, extreme ambient air temperatures or dry and strong winds (Burondkar et al., 2000; Singh et al., 2005; Hagemann et al., 2014, 2015). In plants these factors generally reduce the auxin efflux from as well as the carbohydrate influx to the fruitlet, thus the demand of the growing fruitlet is not sufficiently matched by its supply (Wünsche and Ferguson, 2005; Estornell et al., 2013). This was shown for example in litchi, where branch girdling and defoliation, clearly limiting the carbohydrate supply to the fruitlets, resulted in a decrease of fruitlet auxin concentration which in turn led to abscission (Kuang et al., 2012). This result supports the theory for mango that a reduced basipetal transport of seed-derived auxin through the pedicel (Chacko et al., 1970; Prakash and Ram, 1984; Roemer et al., 2011) and the subsequently increased sensitivity for ethylene in the pedicel abscission zone (AZ) induces fruitlet abscission (Estornell et al., 2013).

Ethylene is perceived by binding to two sub-families of specific ethylene receptors, which control a downstream signal cascade (see reviews of Binder, 2008; Stepanova and Alonso, 2009). Five ethylene receptors have been identified in the model plant Arabidopsis (*Arabidopsis thaliana* (L.) HEYNH.; Binder, 2008) and homologous genes were subsequently described for several crop plants, e.g., six receptors in tomato (Alexander and Grierson, 2002), nine in apple (Ireland et al., 2012), and at present two in mango (Martínez et al., 2001; Ish-Shalom et al., 2011). Based on assessing the triple-response to varying degrees of ethylene perception of Arabidopsis mutants, it was found that a malfunction of one or more receptors can mostly be compensated by the other receptors, however, double mutants of the receptors *ETHYLENE RESISTANT 1* (*AtETR1*) and *ETHYLENE RESPONSE SENSOR 1* (*AtERS1*) exhibit the most severe deficiencies (Binder, 2008). The plant response to ethylene is regulated by receptor specific elements, as for example the *REVERSION-TO-ETHYLENE SENSITIVITY 1* (*AtRTE1*) that exclusively modulates the function of the *AtETR1* (Shakeel et al., 2013) or by receptor-receptor interaction through building homo- and heterodimers or clusters of higher complexity (Gao et al., 2008). These experiments on receptor functionality led to the development of a hierarchical model resulting in *AtETR1* and *AtERS1* being the predominant receptors. Specifically, Patterson and Bleecker (2004) showed in ethylene-insensitive *etr1-1* Arabidopsis mutants that ETR1 delays abscission by reducing the enlargement of the proximal cells within the separation layer. In this context, it is important to note, that O'Malley et al. (2005) showed a positive and linear correlation between ^14^C-ethylene binding activity and the transcript level of ethylene receptors in Arabidopsis and suggested a similar correlative relationship between the transcript and protein level of ethylene receptors. Given the numerous regulatory mechanisms of the ethylene response, it is remarkable that fruitlet and mature fruit abscission seem always associated with a strong upregulation of *ERS1* but not of *ETR1* in pedicels of mango (Ish-Shalom et al., 2011), orange (John-Karuppiah and Burns, 2010), peach (Rasori et al., 2002), and apple (Dal Cin et al., 2008).

Ethephon (2-Chloroethylphosphonic acid) is an ethylene releasing chemical and commonly used to induce thinning of fruitlets or to facilitate the fruit harvesting process (Dennis, 2000; John-Karuppiah and Burns, 2010; Ish-Shalom et al., 2011). In the presence of ethylene, the cells within the fruit pedicel AZ produce cell wall degrading enzymes, thereby inducing the disintegration of the separation layer in the AZ and ultimately leading to the detachment of the fruit (Leslie et al., 2007). Ethephon has previously been used to study the regulation of the mango ethylene receptors *MiERS1* and *MiETR1* during the fruitlet abscission process in laboratory-based experiments (Ish-Shalom et al., 2011). Consequently, the aim of the present study was to investigate the physiological and molecular mechanisms of ethephon-induced fruitlet abscission in mango under field conditions. In particular, emphasis was given on analyzing carbohydrate concentration, polar auxin transport (PAT) capacity and the transcription of ethylene receptors of individual fruitlets and pedicels before and after ethephon spray applications. Moreover, new ethylene receptor versions were identified and their expression patterns interpreted.

## Materials and methods

### Plant material and experimental site

Experiments were conducted over two consecutive fruit growth cycles in 2011 and 2012 in the Tú Nang commune (20°37′0 N, 106°4′60 E) near the township Yên Châu, Province Sơn La, North Vietnam. The mango (*Mangifera indica* L.) trees of the local cultivar “Hôi” were between 10 and 15 years of age. For details on orchard management and phenology see Hagemann et al. (2014).

### Treatments and experimental design

To investigate the physiological and molecular mechanism of fruitlet (pea size; ~4 weeks after full bloom) abscission in mango, fruitlet drop was induced by ethephon spray applications during the critical midseason drop stage. Consequently, there was a greater probability that all fruitlets investigated at each sampling time were at a similar abscission stage. In 2011 and 2012, 12 trees were randomly selected for each of the following treatments: water control and two ethephon (Flordimex 420, Spiess Urania, Germany) treatments at a concentration of 7200 ppm (ET7200) and 600 ppm (ET600). The latter treatment was applied in 2012 to compare the results to those of Ish-Shalom et al. (2011). All treatments were sprayed to run-off with 5 ppm surfactant (Ethalfix® Pro, Syngenta, Switzerland) using a low-pressure handhold sprayer (Gloria, Typ 133, Witten, Germany). For each experimental tree, healthy appearing panicles were randomly tagged at 1 week after full bloom (≥ 90% of all panicles are at least to 80% flowering). For each treatment, six trees with 10 panicles each were used for assessing fruit drop, whereas six trees with 40 panicles each were used for taking fruit samples.

### Fruitlet drop assessment and sampling

**Figure 1:**
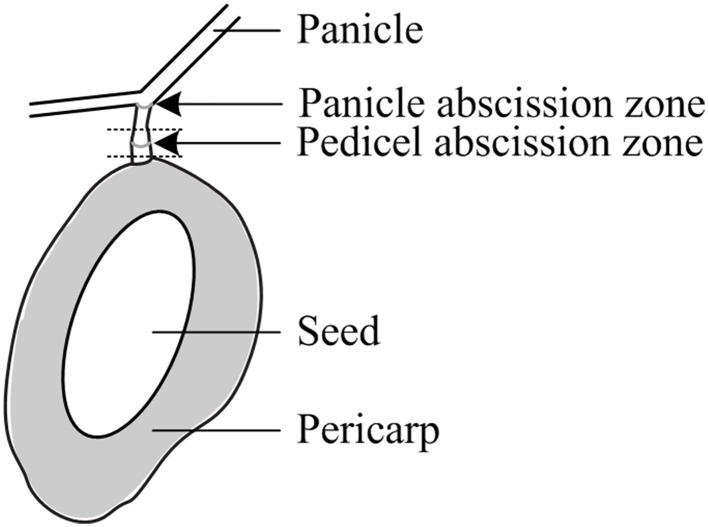
**Schematic diagram of a mango fruitlet with the pedicel and panicle abscission zones**. The dotted lines indicate the tissue with the pedicel abscission zone used for sampling and various analyses.

Fruit retention was recorded every 2 days for the first 4 counting dates and weekly thereafter and expressed as the average fruit number of all initially tagged panicles. Sampling for gene expression and carbohydrate analysis commenced about 2 days (2 ± 1) prior to treatment and continued 1, 2 (only in 2012), and 3 days after treatment (DAT). At each sampling day, 12 fruitlets (averagely two fruitlets from one panicle per tree) were collected for each treatment at noontime. Fruitlet detachment force (FDF) was determined with a gauge (PCE-FM50, Germany) by fixing an individual fruitlet in a customized bracket, holding the entire panicle in position while concomitantly pulling the fruitlet until detachment. In addition, the location of the detachment at the AZ or along the pedicel was recorded and the diameter, length, and weight of each sampled fruitlet as well as the pedicel diameter at mid-position were measured. Each fruitlet was then cut in half and the seed was scored either healthy or degenerated when symptoms of degradation, discoloration, or shrivel were noticed. From each fruitlet, the following parts were sampled for analysis (Figure 1): (1) a 4 mm long pedicel fragment, including the AZ, for gene expression analysis, and fruitlet pericarp for (2) gene expression and (3) carbohydrate analyses. All samples were immediately snap frozen in liquid nitrogen and stored until further processing at −80°C for gene expression analysis and at −30°C for carbohydrate analysis.

Sampling for polar auxin transport (PAT) assay were taken at 2 days (2 ± 1) prior to treatment and at 2 DAT in 2011, whereas at 1 and 3 DAT in 2012. At each sampling day, six panicles (one panicle per tree) were collected per treatment at noontime. The cut end of each panicle was placed in a falcon tube filled with water and transported in sealed styrofoam boxes to the laboratory within 2 h of sampling. Two fruitlets per panicle served for taking records of diameter, length, and weight as well as pedicel diameter at mid-position. The AZ was sampled by cutting 4 mm to either side of the AZ with two parallel mounted razor blades and processed as described in Section Polar Auxin Transport Assay.

### Gene analysis

#### RNA extraction and cDNA synthesis

Frozen fruitlet pedicels and pericarp were ground in liquid nitrogen to fine powder. Total ribonucleic acid (RNA) was extracted from 100 mg subsamples with the MasterPure Plant RNA Purification Kit (Epicentre, USA), following the manufacturer's recommendations. In addition, to reduce the phenolic compounds from the fruitlet pericarp, polyvinylpolypyrrolidone was added in a first step of the extraction process. Genomic desoxyribonucleic acid (DNA) was eliminated with DNaseI and this was subsequently tested by polymerase chain reaction (PCR). RNA samples were stored at −80°C until complementary DNA (cDNA) was synthesized using the TaqMan Reverse Transcription Kit (Applied Biosystems, USA) and following the protocol of the manufacturer. For cDNA synthesis 500 ng of total RNA was used for each reaction. cDNA quality was tested by quantitative real-time PCR (qPCR), using a Rotor-Gene 6000 cycler (Corbett, Australia) with the following conditions: initial denaturation (3 min; 95°C); 40 cycles of denaturation (20 s, 95°C), annealing (20 s, 58°C), and extension (20 s, 72°C); followed by a melt curve from 60°C to 99°C in 0,5 K steps.

#### Gene identification

Specific primers for *MiETR1* were designed (Genbank ID: AF227742.1; Table 1). Conserved regions of *ERS*-like sequences from woody plants and Arabidopsis were identified by alignments to design degenerate primers. Nested PCRs were performed to verify sequence specificity before cloning. The PCR products were then ligated into the pGEM-T vector (Promega, VIC, Australia) following the manufacturer's recommendations. After blue-white selection, a colony PCR with gene specific primers (Table 1) was performed to verify positive clones for subsequent plasmid extraction (QIAPrep Miniprep, Qiagen, Germany) and sequencing (GATC, Germany). Using degenerate primers to identify the homolog to the Arabidopsis *AtERS1*, three different versions of mango *ERS1* were detected and confirmed by sequencing: a version with the full length sequence (*MiERS1*) that is comparable to the *AtERS1*, a medium sized *MiERS1m* with a length of 1203 nucleotides, and a short *MiERS1s* with a length of 561 nucleotides. The sequences were confirmed to be *MiERS1*-like by BLAST search using the NCBI online tool (http://blast.ncbi.nlm.nih.gov) and following the recommendations of Samach (2012).

**Table 1:** **Primers specific for mango genes used for quantitative real-time PCR analysis**.

**Gene**	**Forward primer (5′–3′)**	**Reverse primer (5′–3′)**	**Amplicon size (bp)**
*MiACT*	CCCTGAAGAGCACCCA	AGTTGTACGACCACTGGC	156
*MiUBI*	AAGATCCAGGACAAGGAGG	GGACCAGGTGGAGCG	125
*MiTUB*	ATCAACTACCAGCCACC	CCTTCCTCCATACCCTCAC	184
*MiETR1*	CCAAGGAGAATTGCATGAG	GGCAGCTTGCTCCTC	141
*MiERS1*	TGGCGACAAGAAACGACTG	GCCAGTCTCTTGAAGACTC	116
*MiERS1m*	GCGCTGTAATGAACCATGA	TCTTTGGTATCGTGTTGTC	151
*MiERS1s*	TCTAGTGTCATGTCTAACTGC	GTGCTACCTTTGTCAAGC	115

*β-ACTIN (MiACT), UBIQUITIN (MiUBI), α-TUBULIN (MiTUB), ETHYLENE RESISTANT 1 (MiETR1), ETHYLENE RESPONSE SENSOR 1 (MiERS1), and the two MiERS1 versions MiERS1m and MiERS1s. (Mangifera indica abbreviated as Mi)*.

#### Gene expression studies

The transcription levels of *MiETR1* and the three versions of *MiERS1* were analyzed by qPCR. The efficiency of each primer pair was determined with DART tool (Peirson et al., 2003). Primer specificity was confirmed by melt curve analyses for each individual run and by sequencing of the resulting amplicons. Relative expression of the target genes was analyzed with the efficiency corrected ΔΔCt-method using the DART tool (Peirson et al., 2003). A pool-sample, composed of 1 μl cDNA, was used in each run as a reference for the relative gene expression and as a standard for the different runs. Three potential reference genes, β*-ACTIN* (*MiACT*), α*-TUBULIN* (*MiTUB*), and *UBIQUITIN* (*MiUBI*), were evaluated for their expression stability in the pericarp and pedicel from control and ET7200. *MiACT* was selected as reference gene because it revealed the highest expression stability based on the analysis with the BestKeeper tool (Pfaffl et al., 2004).

### Analysis of soluble carbohydrates

The concentration of fruit soluble carbohydrates was analyzed for all fruitlets that were used for gene expression studies in 2012. Individual fruitlets were ground to a homogenous powder under liquid nitrogen with an impact ball mill (CryoMill, Retsch, Germany). A subsample of 50 mg was taken and re-suspended in 950 μl bi-distilled water, diluted 1:4 and vortexed thoroughly for 1 min. The debris was removed by centrifugation (5 min, 18.000 rcf, 20°C) and 750 μl were collected from the supernatant. Because of the high content of organic acids in the sample, which are disturbance variables in the analytical process, acids were removed from the sample fraction with a strong anion exchange column (Strata-X-A 33u, Phenomenex, CA, USA). Therefore, the columns were pre-conditioned with 8 ml of 0.1 M sodium hydroxide followed by 2 ml of water. The sample was then transferred to the column, eluted with 3 ml water and concentrated to a dry pellet with a rotary evaporator set-up (RC1022, RVT4104, VLP120; Thermo Fisher Scientific Inc., MA, USA). The pellet was re-suspended in 600 μl of water, filtered through a nylon filter with a pore size of 0.45 μm (Wicom, Germany) and injected into the high performance liquid chromatography (HPLC) sampler (Bischoff, Germany). The HPLC setup consisted of a guard column, Hamilton PRP-X400, and a main column, Hamilton HC-75 Ca^2+^ (Hamilton, NV, USA), connected to a refractometric detector (Model 8120; Bischoff, Germany). The carbohydrate separation was done isocratically with bi-distilled water as mobile phase facilitated by two HPLC-pumps (HPLC-Compact-Pump 2250, Bischoff, Germany). The analysis conditions were 80°C at a flow rate of 1.2 ml min^−1^. The amounts of glucose, fructose, and sucrose were quantified using respective standards (Sigma-Aldrich, MO, USA).

### Polar auxin transport assay

To assess the basipetal (polar) auxin transport, the basal end of the fruitlet pedicel was placed onto 96 well-microplates (Greiner bio-one, Germany). Each well-contained 300 μl solidified buffer with 0.05 M 2-(N-morpholino) ethanesulfonic acid (MES), adjusted to pH 5.2, and 1.2% Agar-Agar. A donor block with a volume of 50 μl, shaped as concave disc and consisting of MES buffered 1.5% Agar-Agar, was immediately placed onto the apical side of the pedicel. The acropetal auxin transport was also determined by using 12 additional pedicels in reverse orientation in order to measure the non-polar auxin transport. A droplet of 10 μl [^3^H]-IAA (indole-3-acetic acid labeled with tritium at the 5′ carbon atom of the indol ring with a specific activity of 962 GBq mmol^−1^; Amersham plc, UK) was applied into the cavity of the donor block. Each plate was placed in a dark box with 100% relative humidity and incubated for 8 h at 25°C. After the incubation, the donor block, the pedicel, and the agar of the receiving well (receiver block) were placed into different plastic scintillator vials and stored at −20°C until extraction. For extraction 2 ml of scintillation liquid (Quickzint 212, Zinsser Analytic, Germany) was added to each vial and the samples were incubated at room temperature for 10 days on a rotary shaker at 200 rpm. Thereafter, the [^3^H]-IAA activity as disintegration per minute (dpm) was measured with a liquid scintillation counter (Tri-Carb 3110 TR, PerkinElmer, USA) for 5 min.

### Statistical analysis

The effects of the ethephon treatments on the expression level of ethylene receptors and the concentration of soluble fruit carbohydrates were evaluated by pairwise comparison of the means at a probability level of *p* ≤ 0.05 and the Fisher's least significant difference (LSD) (SAS 9.3; SAS Institute Inc., NC, USA). Model assumptions (normality and variance homogeneity) for the analysis of variance (ANOVA) were checked by examining the residual plots. For analysis of the ethylene receptor expressions, a transformation with the common logarithm was used to stabilize the variance at high expression levels (Rocke and Durbin, 2001), however, the untransformed means are presented in the figures. The results of the PAT experiment and of the FDF measurements did not meet the assumption of variance homogeneity, thus an ANOVA based on ranks (Dunn's post-test) was used to identify differences between treatment groups. In all models various covariates were tested for significant influences on treatment effects.

## Results

### Ethephon induced fruitlet abscission

**Figure 2:**
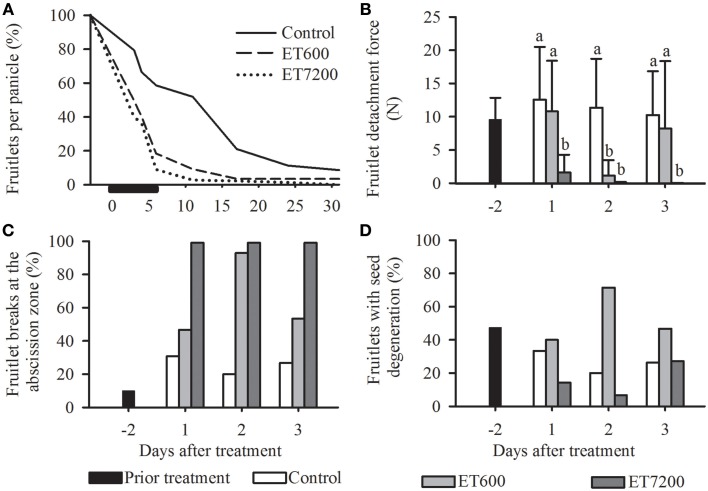
**The effect of the ethephon treatment 600 ppm (ET600) or 7200 ppm (ET7200) spray applications on average (A) fruitlet retention, (B) fruitlet detachment force of fruitlets detaching at the abscission zone or along the pedicel, (C) percentage of fruitlet detachment at the abscission zone (the remainder to 100% are fruitlets detaching along the pedicel) and (D) seed degeneration in comparison to the control at 1, 2, and 3 days after treatment**. **(A)** Horizontal black bar indicates time until 95% of the fruits have abscised in response to ET7200. **(B)** Homogeneous subgroups with no significant difference (*p* ≤ 0.05) are indicated by same letters. Error bars show standard deviation.

Ethephon was used to induce abscission of fruitlets at pea size stage in midseason, allowing the analysis of specific molecular and physiological parameters throughout the process of abscission. Both ethephon concentrations induced an immediate and a much stronger fruitlet abscission than the control treatment (Figure 2A). However, 95% of all fruitlets abscised within 8 days after ET7200 application, whereas it required 6 additional days for ET600 treated fruitlets to reach this level. It is important to note that while ET7200 defruited completely all panicles within 1 month, the ET600 resulted in 2% fruitlets per panicle (Figure 2A). The FDF was significantly reduced by approximately 85% in the ET7200 at 1 DAT and in the ET600 at 2 DAT, respectively, when compared to the control (Figure 2B, Supplementary Figure 1). The FDF in the ET7200 remained extremely low at 2 DAT and was zero at 3 DAT, whereas in the ET600 at 3 DAT it was similar to that of controls (Figure 2B). While all ET7200 treated fruitlets detached at the AZ, a close to 100% abscission at the AZ occurred only at 2 DAT for ET600 fruitlets (Figure 2C). This corresponds in all cases with extremely low FDF values (Figures 2B,C). However, the ET600 application detached only about 50% fruitlet at the AZ at 1 and 3 DAT, which corresponds with relatively high FDF values due to higher detachment forces needed to pull-off the remaining 50% fruitlet somewhere along the pedicel. In contrast, approximately 30% of the controls detached at the AZ, thereby about 70% broke at different locations of the pedicel (Figure 2C). These results are in good agreement with the findings in the previous year (2011), specifically, an ET7200 induced continuous decrease of FDF to zero concomitantly with an increase in fruitlet detachment at the AZ to 100% at 3 DAT (Supplementary Figure 1). Overall, about one third of all fruitlets evaluated showed visible symptoms of seed degeneration; however, this did not seem to be related to the ethephon treatments or FDF (Figure 2D).

### Expression of ethylene receptors in the pedicel

**Figure 3:**
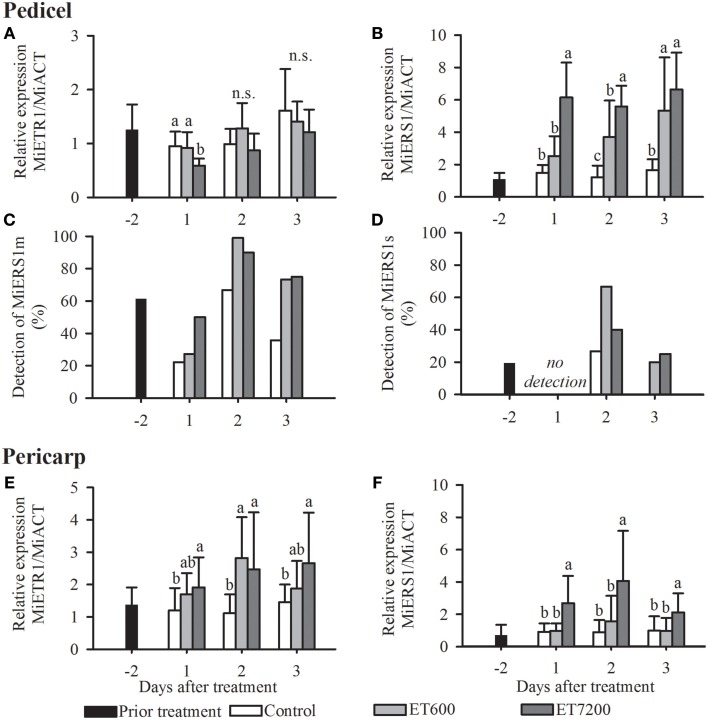
**Expression of the ethylene receptors in the pedicel (A–D) and pericarp (E,F) of pea sized mango fruitlets in response to the ethephon treatment 600 ppm (ET600) and 7200 ppm (ET7200) in comparison to the control at 1, 2, and 3 days after treatment**. **(A)** Expression of *MiETR1* and **(B)** *MiERS1* in the pedicel, **(C)** detection of transcription of *MiERS1m* and **(D)** *MiERS1s* in the pedicel, and **(E)** expression of *MiETR1* and **(F)** *MiERS1* in the pericarp. Homogeneous subgroups with no significant difference (*p* ≤ 0.05) are indicated by same letters. Error bars show standard deviation.

Both ethephon treatments led to a specific receptor transcription pattern in the pedicel, with little response of *MiETR1* and a strong upregulation of *MiERS1* (Figures 3A,B, Supplementary Figure 2). *MiETR1* was not significantly regulated by ET7200, except at 1 DAT in 2012 (Figure 3A, Supplementary Figure 2). In contrast, the expression of *MiERS1* shows a strong response to both ethephon concentrations. ET7200 led to a six and three times higher expression level at 1 DAT in 2011 and 2012, respectively, compared to the control (Figure 3B, Supplementary Figure 2B). The ET7200 induced *MiERS1* upregulation remained higher than the control at the following sampling days, although this was not significant at 3 DAT in 2011 (Figure 3B, Supplementary Figure 2B). The ET600 led to an increasingly stronger *MiERS1* transcription, with a significant *MiERS1* upregulation at 2 and 3 DAT but not at 1 DAT unlike in the case of the ET7200 (Figure 3B).

Three homologs of the Arabidopsis ethylene receptor *AtERS1* have been identified. According to a BLAST analysis all three *MiERS1* versions are highly similar (identity values of 98–99%) to the two full length *MiERS1* GenBank accessions (JN851132.1, JF323582.1). These two accessions derived from the cultivar “Kent,” thus the 1–2% sequence differences are likely a result of a few nucleotide polymorphisms between the cultivars “Hôi” and “Kent.” The “Hôi” *MiERS1* full length has a coding sequence of 1890 nucleotides while the other versions, *MiERS1m* and *MiERS1s* are shorter with 1203 nucleotides and 561 nucleotides, respectively. In contrast to the *MiERS1*, which was detected in all samples (100%), transcripts of *MiERS1m* and *MiERS1s* could only be detected in a much reduced number of samples, although *MiERS1m* was more frequently detected than *MiERS1s* (Figures 3C,D). Nevertheless, transcripts of both shorter receptor versions were detected more often in pedicels of treated fruitlets than in controls (Figures 3C,D). The regulation of *MiERS1m* and *MiERS1s* in the pedicel appears to be erratic, therefore a statistical analysis was not possible (Supplementary Figure 3).

### Expression of ethylene receptors in the fruitlet pericarp

The two receptors *MiETR1* and *MiERS1* were expressed in the fruitlet pericarp with a similar timely pattern in both experimental years (Figures 3E,F, Supplementary Figure 4). Both receptors were significantly upregulated at all DAT following the ET7200 application compared to the control. In contrast, the ET600 led to a significant upregulation of the *MiETR1* only at 2 DAT (Figure 3E, Supplementary Figure 4A), while the transcription level of *MiERS1* was similar to that of the controls at all sampling dates (Figure 3F, Supplementary Figure 4B). Both short versions of *MiERS1* were rarely detected in the fruitlet pericarp (data not shown) and consequently analysis of these receptor versions was not further pursued.

### Polar auxin transport capacity

**Figure 4:**
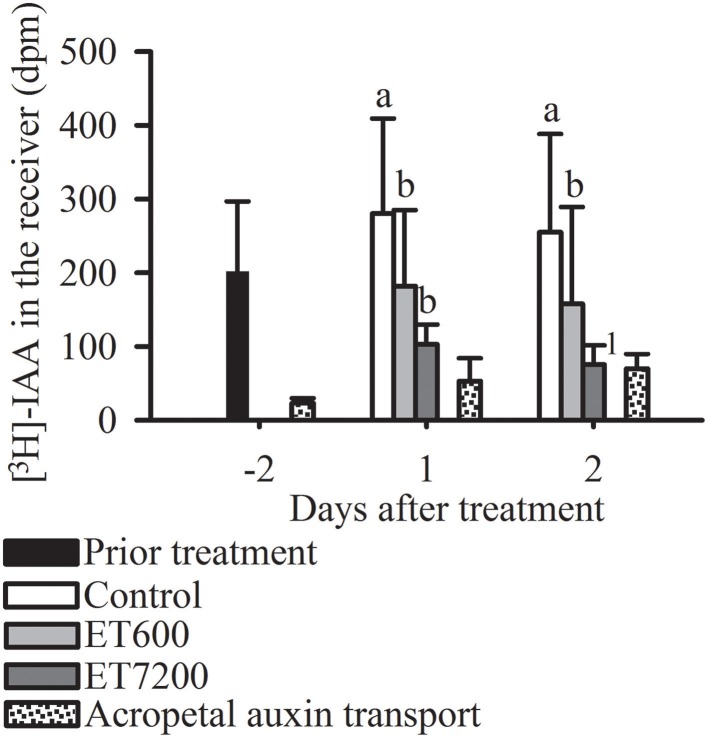
**Polar auxin transport (PAT) capacity through the pedicel of pea sized fruitlets**. Detection of [^3^H]-IAA in the receiver block in response to the ethephon treatment with 600 ppm (ET600) or 7200 ppm (ET7200) in comparison to the control at 1 and 2 days after treatment. Homogeneous subgroups with no significant difference (*p* ≤ 0.05) are indicated by same letters. Error bars show standard deviation; dpm, disintegrations per minute. ^1^sample size (*n* = 3) was too small to perform a statistical test.

The non-polar, acropetal transport capacity of 40 ± 20 dpm was always significantly lower than the PAT capacity of pedicels from control fruitlets (Figure 4). Both ethephon concentrations effectively decreased the PAT capacity of the pedicel at each sampling time (Figure 4, Supplementary Figure 5); however, ET7200 reduced the PAT capacity to a greater extent than the ET600.

### Analysis of soluble carbohydrates

**Figure 5:**
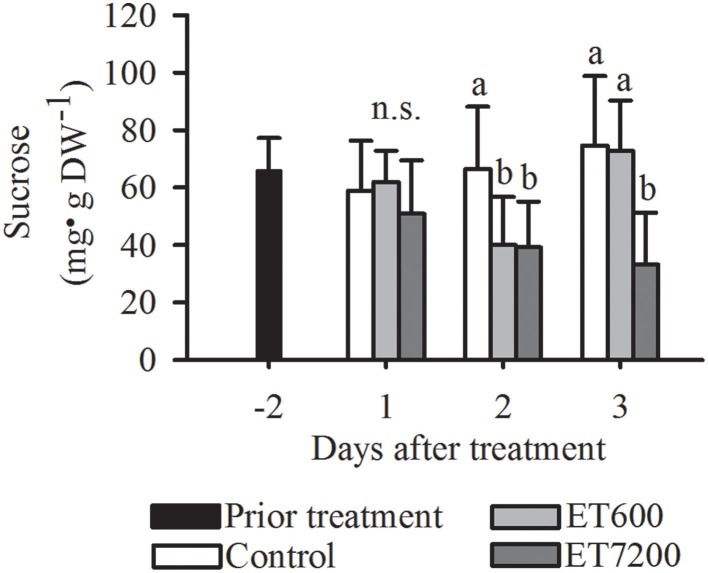
**Sucrose concentration of pea sized fruitlets after the ethephon treatment 600 ppm (ET600) or 7200 ppm (ET7200) in comparison to the control at 1, 2, and 3 days after treatment**. Homogeneous subgroups with no significant difference (*p* ≤ 0.05) are indicated by same letters. Error bars show standard deviation.

Among all the analyzed carbohydrates, a clear response to both ethephon treatments was only found for sucrose, indicated by significantly lower concentrations in treated fruitlets than those in controls at 2 DAT (Figure 5, Supplementary Figure 6). While the sucrose concentration in ET7200 treated fruitlets remained low at 3 DAT, it was not different between ET600 treated fruitlets and controls. Ethephon did not affect the concentration of fructose in the fruitlets (Supplementary Figure 6A). Fruitlet concentration of glucose was significantly increased only at 3 DAT by ET600 compared to the control, whereas was not affected by ET7200 (Supplementary Figure 6B).

## Discussion

The current study supports earlier findings (Malik et al., 2003) that ethephon induced fruitlet abscission in mango is a concentration dependent response: the ET7200 led to a complete loss of fruitlets while approximately 2% of fruitlets were retained in the ET600 at 1 month after spray application (Figure 2A). This clearly indicates that the fruitlet abscission response to ET600 is less pronounced and hence proportionally fewer about-to-abscise fruitlets with a greater FDF value were sampled at 3 DAT when compared to the ET7200.

Irrespective of the treatment applied, low FDF values were symptomatic for fruitlets breaking at the abscission zone (Hagemann et al., 2015, in press) and are indicative of an advanced abscission process. Developmental disorders or nutritional stress during embryogenesis, leading to seed degeneration, was previously suggested as another symptomatic cause of fruitlet abscission (Singh, 1961; Botton et al., 2011). However, despite 30% of the fruitlets containing degenerated seeds, it appeared to be related neither with the point of detachment (data not shown) nor with the ethephon treatments (Figure 2D). Nevertheless, the ET7200 must have induced specific morphological changes at the cellular level within 24 h that led to low FDF values and fruitlets detaching at the AZ, the weakest point along the pedicel (Figures 2B,C). In contrast, this response was only seen 48 h after the ET600. Indeed, microscopy studies of Barnell as early as in 1939 showed for mango that cellular changes within the AZ located between the fruitlet base (flower receptacle) and the pedicel, e.g., meristematic activity and swelling of cell walls, allow a fruitlet to separate with a clean break (Barnell, 1939). Moreover, the action of cell wall degrading enzymes and an increase of turgor pressure are necessary for fruitlet detachment and prior to that, specific genes must have been differentially expressed to induce the AZ (Roberts et al., 2002).

Ethylene receptors were examined as the target genes since the ethylene signaling pathway has been linked to the induction of the AZ and fruitlet abscission (Xie et al., 2013). Of the two ethylene receptors so far described for mango, the *MiETR1* has been reported to be upregulated in the pericarp but not in the pedicel of fruitlets induced for abscission (Martínez et al., 2001; Ish-Shalom et al., 2011). In contrast, *MiERS1* has been reported to be upregulated in the pedicel but not in the pericarp of abscission-induced fruitlets (Ish-Shalom et al., 2011). The current results confirm the findings of Ish-Shalom et al. (2011) that ethephon does not upregulate *MiETR1* but *MiERS1* in the pedicel by using the more sensitive qPCR method instead of the Northern blot (Dean et al., 2002). The about five-times higher concentration than the one used by Ish-Shalom et al. (2011), 1400 vs. 7200 ppm, led to at least 48 h longer upregulation of the *MiERS1* (Figure 3B, Supplementary Figure 2). In general, the *ERS1* responds with an upregulation in the fruitlet pedicels and leaf petioles of different tree crops, including mango, within 24 h of an abscission inducing treatment (Rasori et al., 2002; John-Karuppiah and Burns, 2010; Ish-Shalom et al., 2011). These results corroborate the hypothesis that the role of the *ERS1* in organ abscission is highly conserved in plants. The newly identified short *MiERS1* versions *MiERS1m* and the *MiERS1s* may also be associated with fruitlet abscission because their probability of detection and their expression level were higher in pedicels of ethephon treated and thus abscising fruitlets than in untreated controls (Figures 3C,D, Supplementary Figure 3).

The *MiETR1* upregulation in the pericarp of ethephon treated fruitlets was more pronounced following the ET7200 compared to the ET600 (Figure 3E). The ET600 induced significant upregulation of the *MiETR1* but not of the *MiERS1* in the pericarp, corresponds to the findings of Ish-Shalom et al. (2011). It is important to note that Ish-Shalom et al. (2011) applied a higher concentration of ethephon at a lower temperature (1400 ppm at 20°C) compared to the current study (600 ppm at 29°C), hence both studies are comparable due to the temperature-depending effect of ethephon (Yuan and Burns, 2004). In contrast, the ET7200 led to a significant upregulation of both ethylene receptors in the pericarp of fruitlets from 1 DAT onwards (Figures 3E,F). Thus, the expression pattern of both receptors clearly indicates an ethephon (ethylene) concentration dependent response. It is likely that ET7200 induced a greater endogenous autocatalytic ethylene synthesis, which largely contributes to a longer lasting and significantly greater ethylene receptor response. It may also be that the ethylene sensitivity threshold of the AZ is in part maintained through the ET7200 application despite a 50% ethephon degradation within 1 DAT (Domir and Foy, 1978). In the natural abscission process, it is suggested that fruitlet-derived ethylene is synthesized in the pericarp and diffuses to the AZ (Nunez-Elisea and Davenport, 1986; Malik et al., 2003) where it induces the upregulation of ethylene receptors (Stepanova and Alonso, 2009) prior to the induction of the abscission process. These findings lead to the hypothesis that during natural abscission, ethylene receptors are first upregulated in the fruitlet and then in the pedicel (Hagemann et al., in press). Chemical induction of the abscission by ethephon would result in a simultaneous upregulation of ethylene receptors in fruitlets and pedicels (Figure 3).

Another key element of the abscission process is the auxin signaling (Xie et al., 2013), which was expressed as PAT capacity in the present study. Untreated mango fruitlets transported only 5% of the radioactively labeled auxin through an 8 mm long pedicel within 8 h, while it was 38% through 4 mm sweet cherry pedicles within 3 h (Else et al., 2004) and 5–13% through 15 mm lupine hypocotyls within 8 h (Sánchez-Bravo et al., 1992). In mango the vascular system is in close association with resin canals and exudates rich in carbohydrates and phenolic compounds cause a rapid sealing of the cut surface (Joel, 1981; Lima Filho, 2004), thus likely reducing the PAT capacity. However, a sealing of the cut surface was prevented by immediately placing a physiologically-buffered agar block on the cut surfaces. Both ethephon treatments reduced the PAT capacity of mango fruitlet pedicels within 24 h (Figure 4) which supports earlier findings that the transcript of an auxin efflux carrier responsible for the basipetal auxin transport (Friml, 2003) was reduced within 24 h of ethylene treatment (Dal Cin et al., 2009). Experiments with Arabidopsis seedlings showed that ethylene biosynthesis pathway enzymes respond to varying auxin concentrations (Abel et al., 1995), suggesting that a reduced PAT through the pedicel can also induce endogenous ethylene evolution in pedicels and in turn trigger abscission in the AZ.

Carbohydrate deficiency is another plausible cause of fruitlet abscission (Xie et al., 2013), however, few data of carbohydrate concentrations in mango fruitlets during the main fruitlet drop stage at pea to marble size are available. Defoliation experiments with citrus have clearly shown that low sucrose concentration in fruitlets cause fruitlet abscission (Mehouachi et al., 1995) and in agreement with this finding, also low concentration of sucrose in mango fruitlet seem to be related to the abscission inducing treatment (Figure 5, Supplementary Figure 6). Sucrose concentration in the pulp of mature mango fruit ranged from 46 to 114 mg g^−1^ dry weight, depending on cultivar, ripening stage and method used for analysis (Thanaraj et al., 2009). Moreover, it was shown earlier for mango that sucrose is the main translocation carbohydrate in support of fruit growth (Chauhan and Pandey, 1984). It is suggested that the ethephon-induced reduction of sucrose concentration in fruitlets at 2 DAT is triggered by reduced auxin signaling that subsequently reduces the sink strength for carbohydrate import into the fruitlet commencing at 1 DAT (Figure 5).

**Figure 6:**
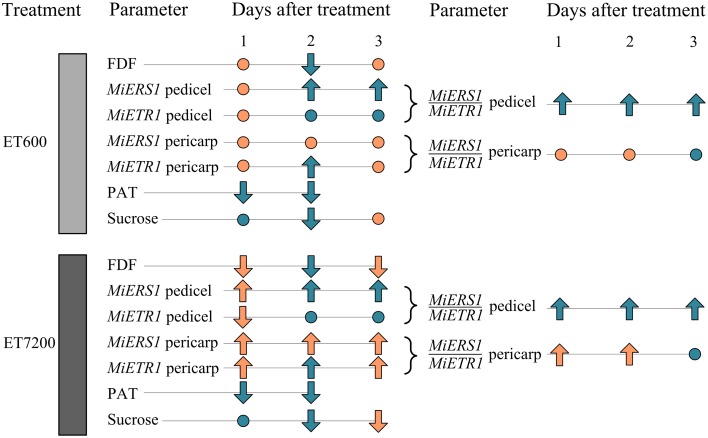
**Overview of the key fruitlet abscission parameters analyzed in this study**. Parameters of ethephon treated fruitlets compared to those of control fruitlets: no significant differences are indicated by a dot, whereas up- or downward pointing arrows indicate significant differences. Different or equal response of the ethephon treatments 600 ppm (ET600) and 7200 ppm (ET7200) in comparison to the control are indicated by orange and cyan colored symbols, respectively. The parameters are: fruitlet detachment force (FDF), gene expression of the ethylene receptors *MiERS1*, and *MiETR1* and their ratios in the pedicel and fruit pericarp, polar auxin transport (PAT), and the concentration of sucrose in the fruit pericarp.

In conclusion, the data suggest that the ethephon-induced fruitlet abscission follows a different sequence of events (Figure 6) compared to the natural abscission process. In the latter case, resource deficiency, e.g., carbohydrate supply limitations for fruitlet growth, or seed degeneration with auxin signaling disruption are primary physiological causes (Xie et al., 2013; Hagemann et al., in press). In contrast, the ethephon-induced fruitlet abscission process responds initially with a reduction of the PAT capacity in the pedicel, followed by an upregulation of ethylene receptors and then a decline in sucrose concentration; physiological markers that were not linked to seed degeneration. Ethephon spray applications at the high concentration caused a faster abscission of mango fruitlets at the AZ than the low ethephon concentration. This might be due to a more rapid saturation of ethylene receptor binding sites in the pedicel by the high ethephon concentration, which presumably also causes a greater autocatalytic ethylene production in the pericarp and the pedicel.

An alternative explanation is provided by Dal Cin et al. (2005) who first suggested that a greater *ERS1/ETR1* ratio in both the pedicel AZ and the fruit cortex (pericarp) is a decisive trigger for fruitlet abscission during the midseason drop stage in apple. This notion was also suggested for mango (Ish-Shalom et al., 2011); however, specific evidence is provided in the present study with higher *MiERS1/MiETR1* ratios in the pericarp and the pedicel of ET7200-treated fruitlets than those of control fruitlets (Figure 6; Supplementary Table 1). In contrast, ET600 induced an increased *MiERS1/MiETR1* ratio in the pedicel but not in the pericarp, suggesting that the receptor regulation in the pericarp is not the primary determining factor in both ethephon-inducing fruitlet abscission treatments (Figure 6; Supplementary Table 1). However, the 1-day earlier reduction of FDF in the ET7200 than the ET600 might be associated with the higher *MiERS1/MiETR1* ratios in both pedicel and pericarp. Following the *MiERS1/MiETR1* ratio concept, the ethephon-induced fruitlet abscission process commences with a reduction of the PAT capacity and an upregulation of ethylene receptors.

The findings of this study contribute to further our understanding of the regulation of ethylene signaling in horticultural crops. The ERS1 versions might also be able to interact with one another to build receptor clusters as previously described by Gao et al. (2008) for Arabidopsis. It was shown that some receptors and in particular ERS2 and the ETR1 form preferentially heterodimers which in turn can form receptor clusters through the GAF domains (Gao et al., 2008), known as common elements of ethylene receptors necessary for the receptor-receptor interaction (Binder, 2008; Gao et al., 2008). Moreover, it was concluded that a given receptor within the cluster can laterally transmit the signal of detecting an ethylene molecule to neighboring receptors, thereby amplifying the signal and subsequently inducing the ethylene response at even low ethylene concentrations. Liu and Wen (2012) discussed different scenarios of receptor cluster function. Following the model for family 1 receptor clusters with a strong signal output, the identified *MiERS1* versions could lead to reduced ethylene sensitivity when expressed and translated. These cluster functions could partly explain the numerous ethylene-induced plant responses, e.g., the different abscission response to ethylene of leaves and fruit as shown for citrus (John-Karuppiah and Burns, 2010). The findings may also provide new breeding targets for mango as for example selecting genotypes for mutated ERS1 receptors that are less sensitive to ethylene and thereby less prone to fruitlet abscission.

## Author contributions

The authors have drafted and contributed to the concept and design of the work together. Further the authors have critically revised the work for its scientific significance and approved this version of this manuscript for publication. Additionally the specific contributions of the authors were as followed:

**MHH** conducted the sample and data collection in Vietnam, the sample processing, as well as technical and statistical data analysis. He adapted and further developed the HPLC-sample extraction and the qPCR data processing. Further he drafted the publication and adapted the layout.

**PW** developed the concept of the molecular biological part of the work. He established the RNA extraction and analysis including the cloning and sequence evaluation. Further he identified the genes and established the corresponding qPCR analysis.

**MH** supported the establishment of the field trials and orchard management in Vietnam. Further he developed the concept of the polar auxin transport assay including sample extraction, analysis and data interpretation.

**JW** drafted the main concept of the experimental design. He supervised the study and critically revised the outcomes for their overall scientific impact. Together with the corresponding authors he developed the data analysis, data interpretation and the final manuscript.

### Conflict of interest statement

The authors declare that the research was conducted in the absence of any commercial or financial relationships that could be construed as a potential conflict of interest.

## Abbreviations

*ACT*: β-*ACTIN*
AZ: abscission zone
At: *Arabidopsis thaliana*
cDNA: complementary desoxyribonucleic acid
DAT: days after treatment
*ERS1*: *ETHYLENE RESPONSE SENSOR 1*
*ETR1*: *ETHYLENE RESISTANT 1*
ET: ethephon treatment
ET600: ethephon treatment 600 ppm
ET7200: ethephon treatment 7200 ppm
FDF: fruitlet detachment force
IAA: indole-3-acetic acid
Mi: *Mangifera indica*
MES: 2-(N-morpholino) ethanesulfonic acid
PAT: polar auxin transport
qPCR: quantitative real-time PCR
PCR: polymerase chain reaction
rcf: relative centrifugal force
RNA: ribonucleic acid
*RTE1*: *REVERSION-TO-ETHYLENE SENSITIVITY1*
*TUB*: α-*TUBULIN*
*UBI*: *UBIQUITIN*.
