# Supplementary material for: Ethephon induced abscission in mango: physiological fruitlet responses
Source: Front Plant Sci. 2015 Sep 15;6:706. doi: 10.3389/fpls.2015.00706 (PMC4569964; doi:10.3389/fpls.2015.00706)
Supplement: Supplementary file 1 [file DataSheet1.DOCX]

***Supplementary Material***

**Ethephon induced abscission in mango: physiological fruitlet responses**

**Michael H. Hagemann^1*^, Patrick Winterhagen^1^, Martin Hegele^1^ and Jens N. Wünsche^1^**

^1^ Section Crop Physiology of Specialty Crops, Institute of Crop Science, University of Hohenheim, Stuttgart, Germany

*** Correspondence:** Michael H. Hagemann, University of Hohenheim, Institute of Crop Science, Section Crop Physiology of Specialty Crops, Emil-Wolff-Str. 25, Stuttgart, 70599, Germany.

michael@uni-hohenheim.de

## Supplementary Figures


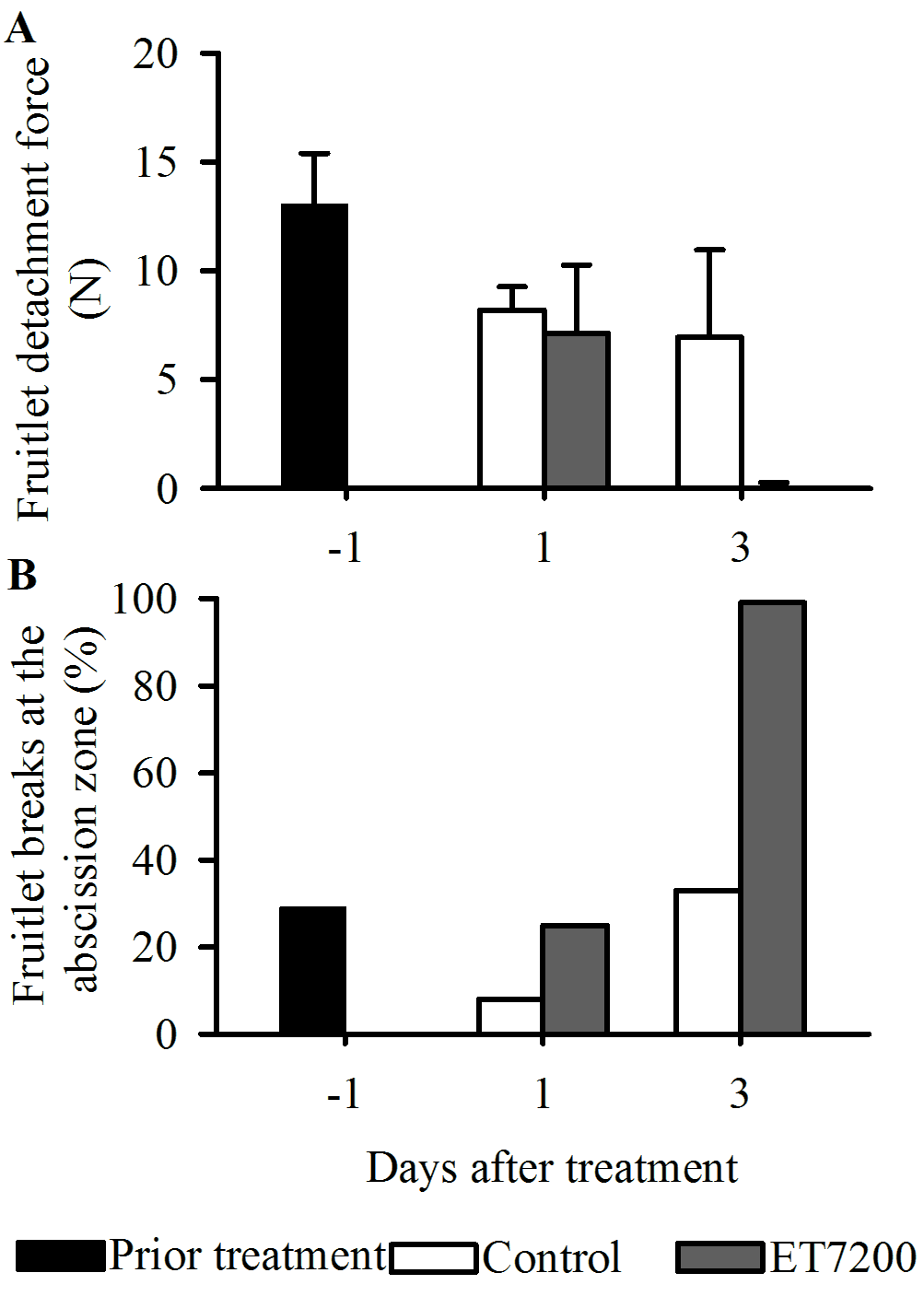


**Supplementary Figure 1.** The effect of ethephon treatment 7200 ppm (ET7200) on average **(A)** fruitlet detachment force of fruitlets detaching at the abscission zone or along the pedicel and **(B)** percentage of fruitlet detachment at the abscission zone (the remainder to 100% are fruitlets detaching along the pedicel) in comparison to the control and at 1 and 3 days after treatment. Error bars show standard deviation. Data from 2011.


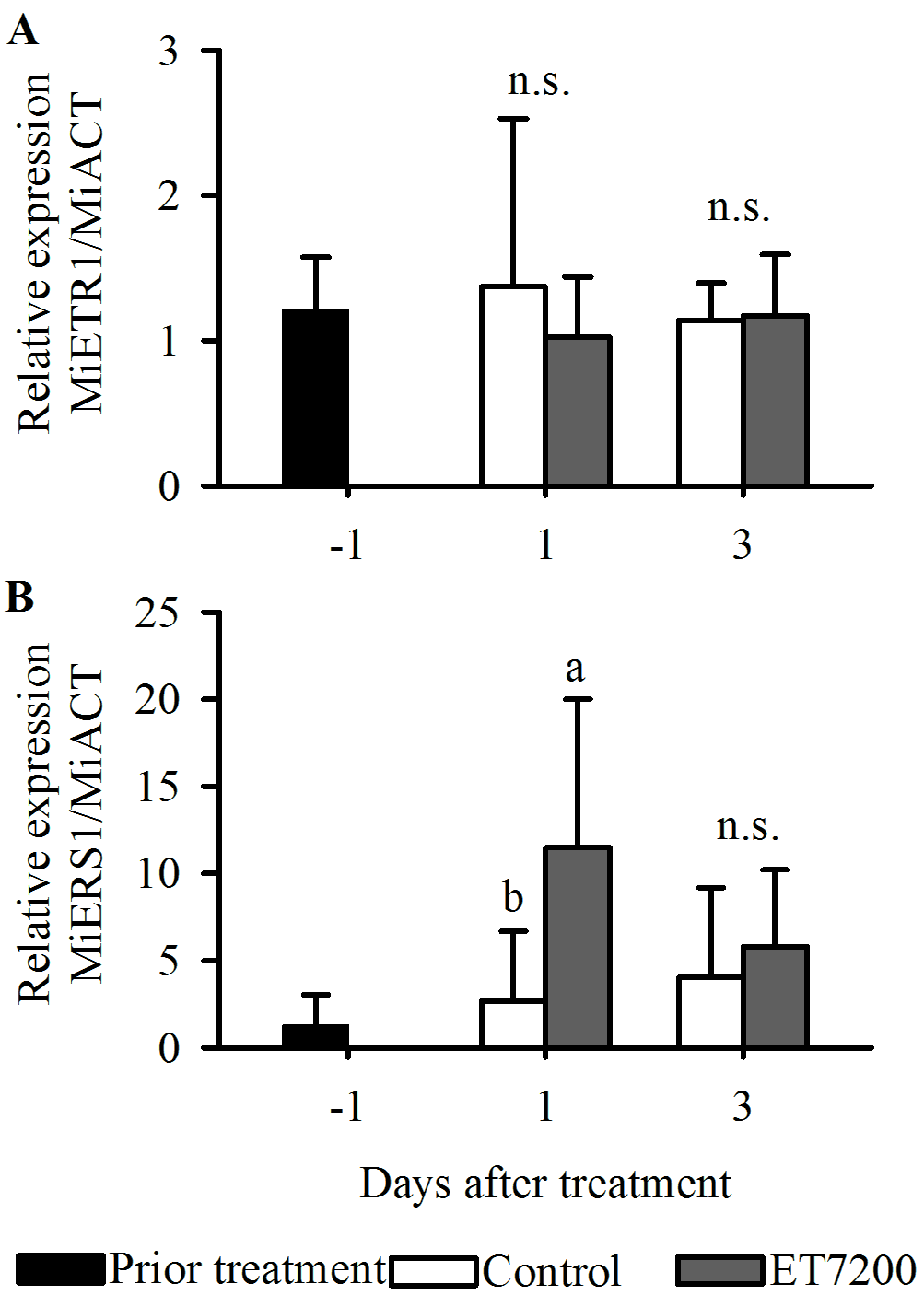


**Supplementary Figure 2.** Expression of the ethylene receptors **(A)** *MiETR1* and **(B)** *MiERS1* in the pedicel of pea sized mango fruitlets in response to the ethephon treatment 7200 ppm (ET7200) in comparison to the control at 1 and 3 days after treatment. Homogeneous subgroups with no signiﬁcant difference (*p ≤ 0.05*) are indicated by same letters. Error bars show standard deviation. Data from 2011.


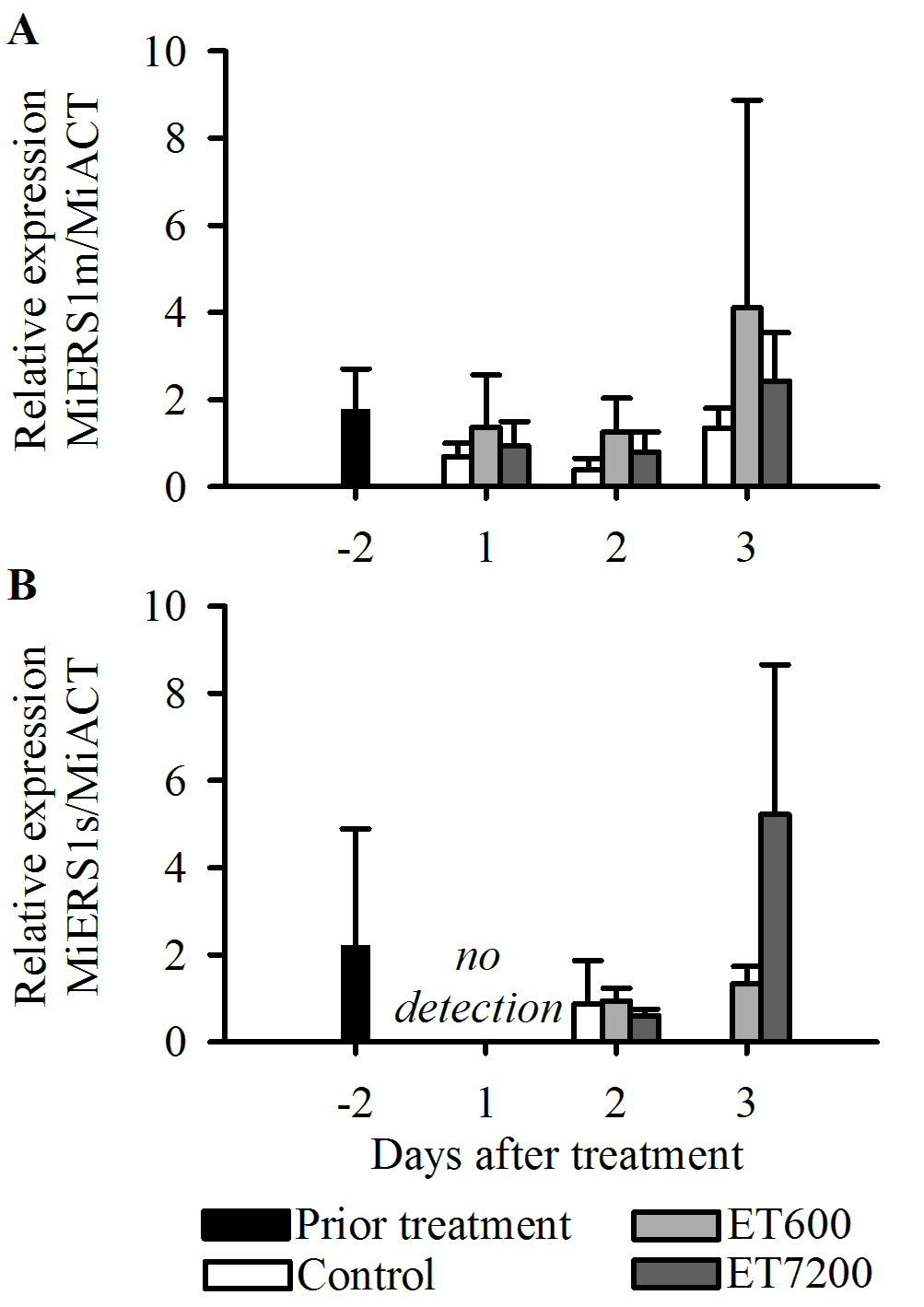


**Supplementary Figure 3.** Expression of short versions of the *MiERS1.* **(A)** *MiERS1m* and **(B)***MiERS1s* in the pedicel of pea sized mango fruitlets in response to the ethephon treatments 600 ppm (ET600) or 7200 ppm (ET7200) in comparison to the control at 1, 2 and 3 days after. No statistical test possible due to highly variable sample size. Missing error bar indicates n=1. Data from 2012.

**
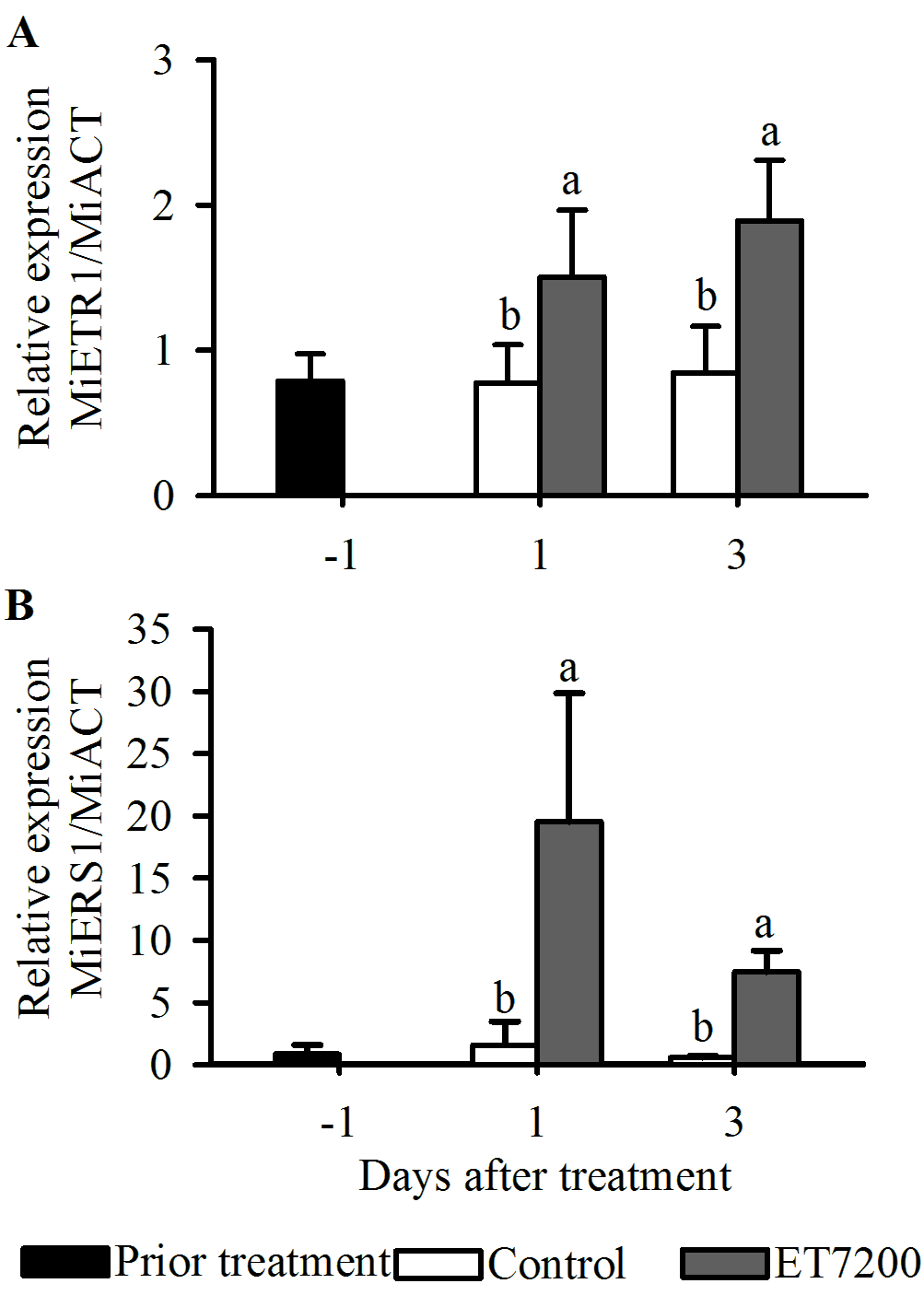
**

**Supplementary Figure 4.** Expression analysis of **(A)** *MiETR1* and **(B)** *MiERS1* in the pericarp of pea sized fruitlets in response to the ethephon treatment 7200 ppm (ET7200) in comparison to the control and at 1 and 3 days after treatment. Homogeneous subgroups with no signiﬁcant difference (*p ≤ 0.05*) are indicated by same letters. Error bars show standard deviation. Data from 2011.


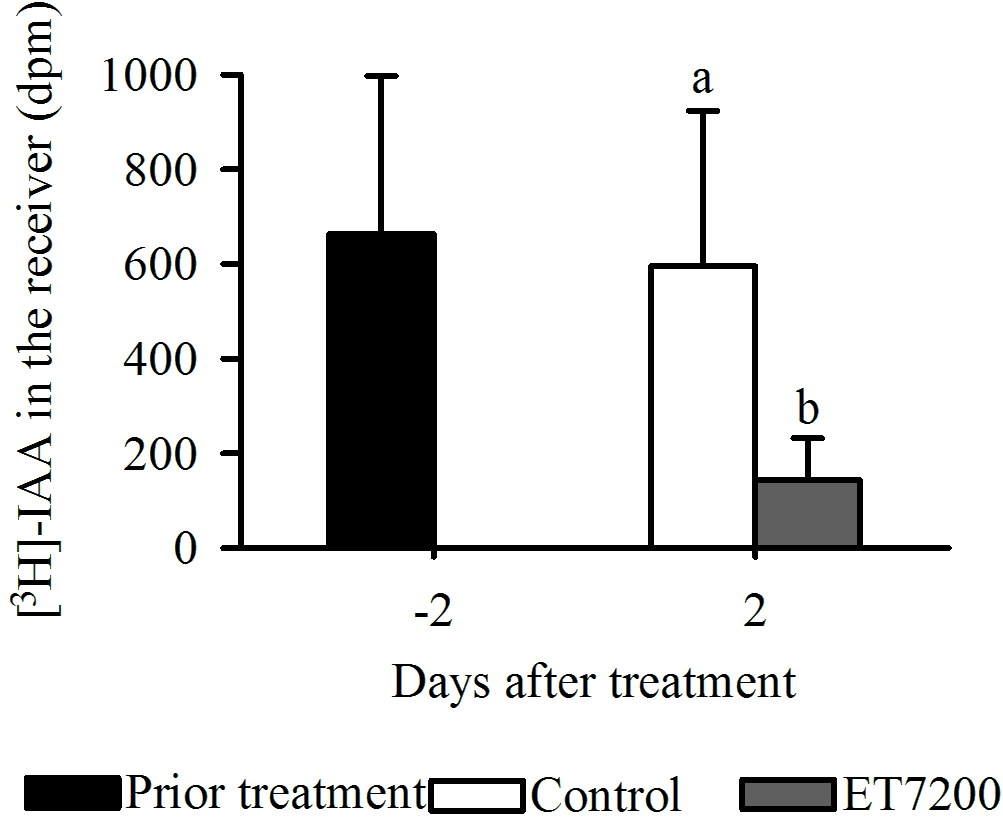


**Supplementary Figure 5.** Accumulated [^3^H]-IAA in the receiver after the ethephon treatment 7200 ppm (ET7200) in comparison to the control at 2 days after treatment. Homogeneous subgroups with no signiﬁcant difference (*p ≤ 0.05*) are indicated by same letters. Error bars show standard deviation; dpm = disintegrations per minute. Data from 2011.


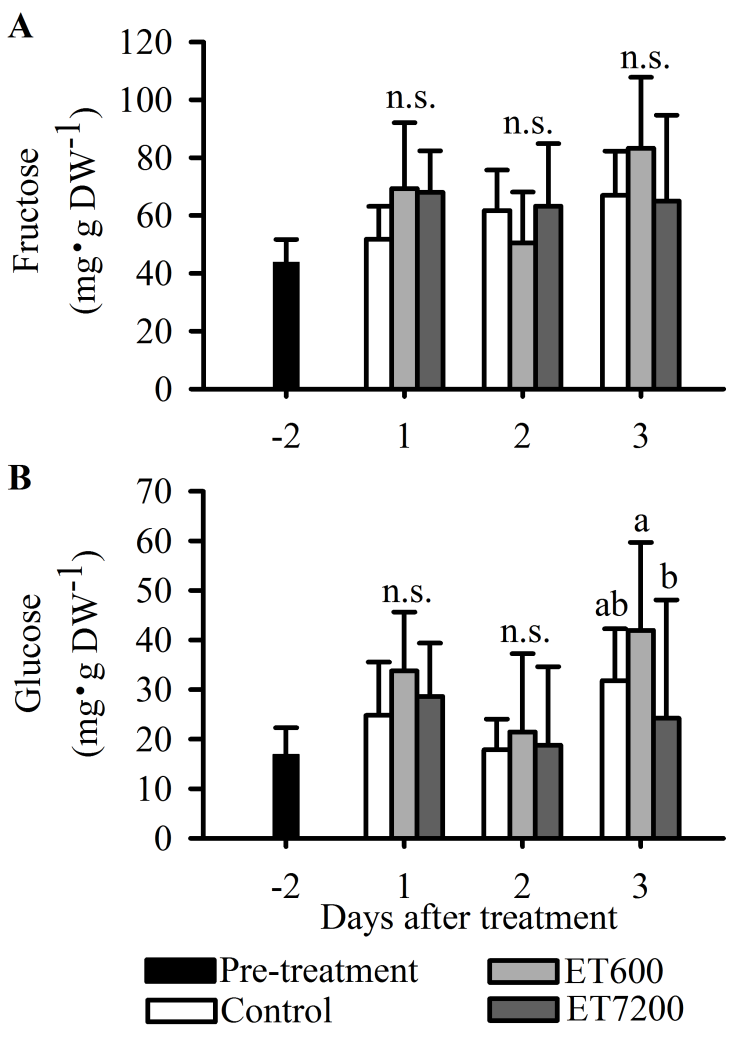


**Supplementary Figure 6.** Concentrations of fructose and glucose of pea sized fruitlets after the ethephon treatments 600 ppm (ET600) or 7200 ppm (ET7200) in comparison to the control at 1, 2 and 3 days after treatment. Homogeneous subgroups with no signiﬁcant difference (*p ≤ 0.05*) are indicated by same letters. Error bars show standard deviation. Data from 2012.

## Supplementary Tables

**Supplementary Table 1.** Ratio of the ethylene receptors *MiERS1* and *MiETR1* after the ethephon treatments 600 ppm (ET600) or 7200 ppm (ET7200) in comparison to the control. Data from 2012.

| Parameter | Treatment | Days after treatment | | |
| --- | --- | --- | --- | --- |
|  |  | 1 | 2 | 3 |
| MiERS1/MiETR1 pedicel | Control | 1.6 | 1.2 | 1.0 |
|  | ET600 | 2.7 | 2.9 | 3.8 |
|  | ET7200 | 10.4 | 6.4 | 5.5 |
| MiERS1/MiETR1 pericarp | Control | 0.8 | 0.8 | 0.7 |
|  | ET600 | 0.6 | 0.6 | 0.5 |
|  | ET7200 | 1.4 | 1.6 | 0.8 |
